# Does age affect perception of the Speech-to-Song Illusion?

**DOI:** 10.1371/journal.pone.0250042

**Published:** 2021-04-19

**Authors:** Hollie A. C. Mullin, Evan A. Norkey, Anisha Kodwani, Michael S. Vitevitch, Nichol Castro

**Affiliations:** 1 University of Kansas, Lawrence, KS, United States of America; 2 University at Buffalo, Buffalo, NY, United States of America; Birkbeck College, UNITED KINGDOM

## Abstract

The Speech-to-Song Illusion is an auditory illusion that occurs when a spoken phrase is repeatedly presented. After several presentations, listeners report that the phrase seems to be sung rather than spoken. Previous work [1] indicates that the mechanisms—priming, activation, and satiation—found in the language processing model, Node Structure Theory (NST), may account for the Speech-to-Song Illusion. NST also accounts for other language-related phenomena, including increased experiences in older adults of the tip-of-the-tongue state (where you know a word, but can’t retrieve it). Based on the mechanism in NST used to account for the age-related increase in the tip-of-the-tongue phenomenon, we predicted that older adults may be less likely to experience the Speech-to-Song Illusion than younger adults. Adults of a wide range of ages heard a stimulus known to evoke the Speech-to-Song Illusion. Then, they were asked to indicate if they experienced the illusion or not (Study 1), to respond using a 5-point song-likeness rating scale (Study 2), or to indicate when the percept changed from speech to song (Study 3). The results of these studies suggest that the illusion is experienced with similar frequency and strength, and after the same number of repetitions by adult listeners regardless of age.

## Introduction

Perceptual illusions occur when our percept does not match what is actually in the environment. Although illusions provide the general public (and zoo animals [2]) with entertainment, perceptual illusions also provide psychologists with a way to examine the limits of our perceptual and cognitive systems, thereby increasing our fundamental understanding of these systems [3, 4].

The Speech-to-Song Illusion is an auditory illusion that occurs when a spoken phrase is presented repeatedly to the listener. After several presentations of the phrase, many listeners report that the phrase appears to be sung, rather than spoken [5]. Importantly, the phrase is not altered during the multiple presentations; it is still spoken, only the percept changes. Subsequent studies observed the illusion in languages other than English (*e*.*g*., German [6]). Further, brain regions associated with speech and music perception exhibited activation when participants perceived the illusion [7], attesting to the robustness of this auditory illusion.

**Fig 1:**
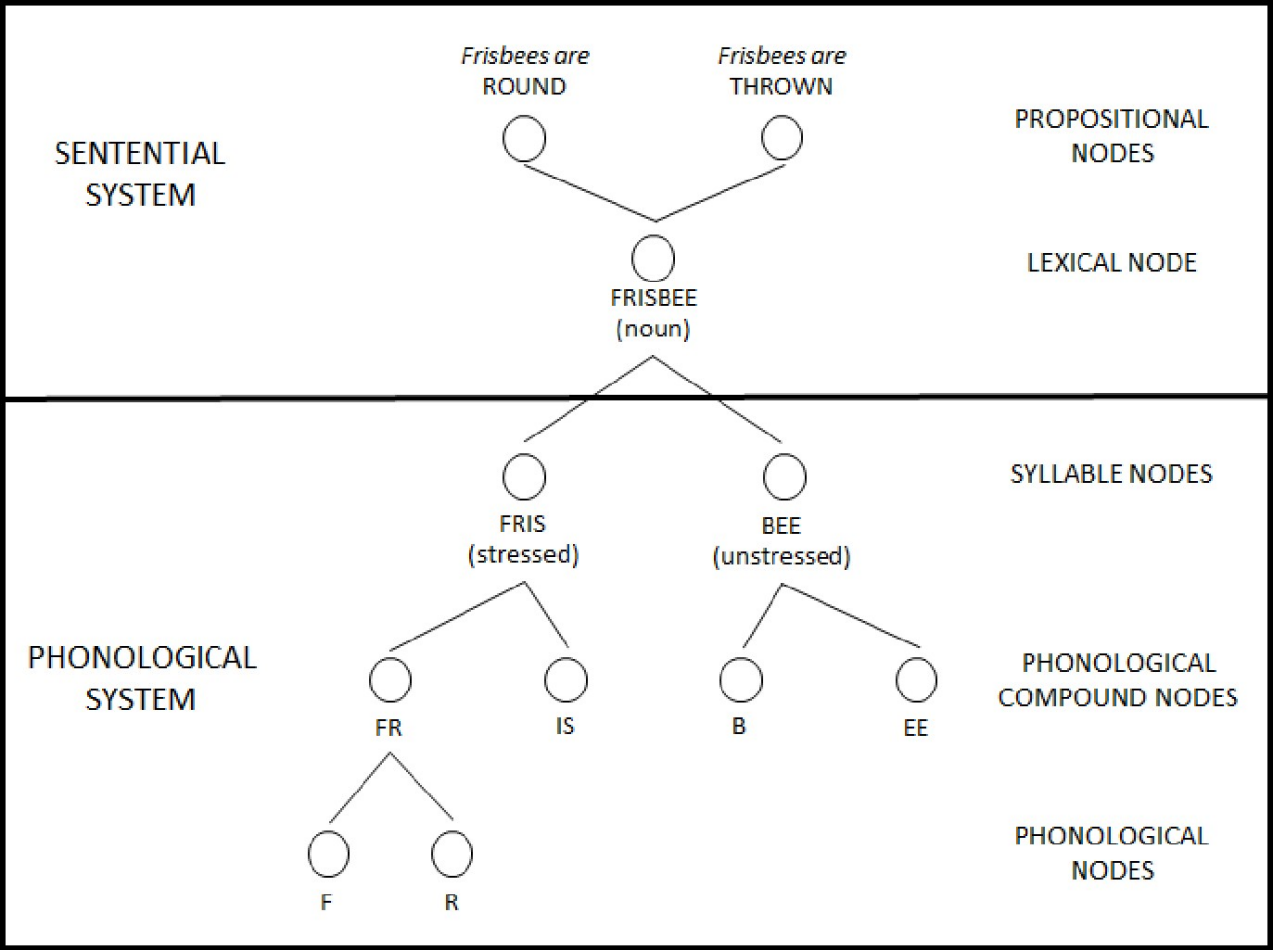
Nodes representing phonemes, syllables, and semantic information associated with the word *frisbee* as it might be represented in Node Structure Theory. Additional higher-level and lower-level nodes have been omitted to simplify the image.

Recent work [1, 8, 9] suggests that the mechanisms—priming, activation and satiation—found in the language processing model, Node Structure Theory (NST), may account for the Speech-to-Song Illusion. NST is a model of language production and perception [10] that contains “detectors” or nodes representing different types of information (*e*.*g*., phoneme, syllable, word; See Fig 1). When sufficient amounts of *priming* (akin to spreading activation in other models) accumulates a node is *activated*, which brings the information represented by that node to conscious awareness [11]. Repeated activation of a node leads to a temporary inability to activate the node again, a state known as *satiation*, which also results in the temporary inability to consciously access the information associated with that node.

In the Speech-to-Song Illusion, the presentation of words in a phrase primes and activates the corresponding lexical nodes, bringing those words to conscious awareness and giving the initial percept of speech [1]. Repeated presentation of the phrase leads to satiation of those lexical nodes, resulting in the loss of the speech percept. However, the repeated presentation of the phrase continues to prime the syllable nodes that comprise the words. Because syllables encode the rhythmic information of language, a rhythmic, song-like percept then emerges [1].

NST has also been used to explain other language-related phenomena, including the tip-of-the-tongue (TOT) state, which is the feeling of familiarity with a word, but a temporary inability to retrieve it completely [11]. TOT states tend to occur more frequently in older adults compared to younger adults [12]. According to NST, aging weakens the connections that exist between nodes, negatively affecting the amount of priming that can be transmitted between nodes [11]. This age-related decrease in the transmission of priming is referred to as the *transmission deficit hypothesis*, and it is thought to lead to the increase in TOT states in older adults [13].

Given that the transmission deficit hypothesis in NST has been used to account for the effects of age in a phenomenon of speech production (i.e., the TOT state), and other studies have observed effects of aging in speech perception [14], we were motivated to examine if the perceptual illusion known as the Speech-to-Song Illusion—where the initial *speech* percept shifts to a song-like percept—is also influenced by age. In addition, age-related differences have been observed in certain visual illusions, with 4–5 year-olds experiencing the Ebbinghaus and Ponzo illusions, but not the rectangle or 3D-cube illusions, whereas seven-year-olds and adults reported experiencing all four of the visual illusions [15]. The observation of age-related differences in visual illusions further motivated us to look for age-related differences in the auditory illusion known as the Speech-to-Song Illusion.

Based on the transmission deficit hypothesis in NST, older adults have more difficulty transmitting priming between nodes, leading to impaired activation of certain nodes [13]. If insufficient amounts of priming are transmitted to the syllable nodes—which encode the rhythmic information of language and are thought to lead to the song-like percept in the Speech-to-Song Illusion—then older adults should be less likely to experience the Speech-to-Song Illusion than younger adults [1]. We conducted three on-line surveys to examine this age-related hypothesis about the Speech-to-Song Illusion.

## Study 1

An increasing number of studies have used a variety of stimuli to examine various aspects of the Speech-to-Song Illusion, making this illusion a well-established perceptual phenomenon [16–24]. To provide a strong test of our age-related hypothesis about the Speech-to-Song Illusion, we attempted to maximize the likelihood that listeners would experience the Speech-to-Song Illusion by using the original stimulus recorded by [25], rather than the variety of stimuli that have been used previously [e.g., 1, 8]. This stimulus phrase is widely-used in demonstrations of the illusion (http://philomel.com/asa156th/mp3/Sound_Demo_1.mp3). We excised from the original sound file the phrase, “sometimes behave so strangely,” and presented it a total of 10 times as has been done in previous studies [e.g., 1]. If there is indeed an age-related difference in the perception of the Speech-to-Song Illusion, then we should be able to observe it even when the canonical stimulus is presented to evoke the illusion.

To quickly collect data from participants with a wide range of ages, we turned to Amazon’s Mechanical Turk (MTurk), a website increasingly being used to conduct research in the field of psychology. Through this website, researchers are able to quickly recruit a large participant pool to engage in simple, streamlined studies and compensate the participants upon task completion. Samples recruited via MTurk are more diverse than samples recruited from the typical American college, and that the data obtained via this on-line method are as reliable as those obtained via traditional methods (*i*.*e*., in-person, laboratory-based studies) [26, 27].

Qualtrics was used to create a short survey for participants to complete. The survey consisted of a written consent form (if participants did not give e-consent, they could not participate in the study), a short, written explanation of the Speech-to-Song Illusion, and the presentation of our edited audio file based on [25] Speech-to-Song Illusion inducing phrase. After listening to the illusion-eliciting stimulus, participants indicated whether or not they experienced the illusion.

### Method

#### Participants

We restricted our recruitment on MTurk to individuals from the United States of America. Participants were paid $0.25 for their time. A group of 100 participants was initially recruited with no other restrictions. After examining the reported ages of that initial group of participants, we wished to obtain a larger number of older adults. Therefore, we recruited 100 more participants and restricted the age to 55 years and older (only 99 participants completed the task before it expired after being posted for 10 days). In total, 199 participants were recruited and completed the survey.

We did not collect data on the gender, handedness, language background, or hearing acuity of the participants. We recognize that age-related hearing loss (i.e., presbycusis) is common [28], however we assumed that each participant had the “volume” setting on their computer (i.e., the setting that controls the amplitude of an audio signal) adjusted to a listening level that was comfortable to their individual preferences.

To screen out bots, individuals who did not attend to the task, or individuals that may have experienced age-related hearing problems that adversely affected speech perception, we excluded participants who responded incorrectly to a question regarding the content of our audio file (*i*.*e*., What phrase was being repeated in the audio file?). This left data from 157 participants ranging in age from 18–81 years to be analyzed. The mean age of all of the participants was 50.82 years (*sd* = 16.00).

#### Procedure

The study was approved by the institutional review board at the University of Kansas and administered through a Qualtrics (December 2020) survey in English. A captcha was presented at the beginning of the survey as an initial screening for bots (i.e., non-human robots that automatically respond to survey questions, or aid humans to respond more rapidly to survey questions [29]). Once written e-consent was received, participants answered several demographic questions: What is your age? Do you have musical experience (have you played an instrument, participated in choir, etc.)? (If yes,) How many years of musical experience do you have (how many years have you played an instrument, participated in choir, etc.)?.

A written explanation of the Speech-to-Song Illusion was then presented on the screen for 30 seconds (and could not be advanced until the timer ran out):

*Illusions occur when our senses incorrectly perceive what is in our environment*. *In this study*, *we are interested in an auditory illusion called the Speech-to-Song Illusion*. *In the Speech-to-Song Illusion*, *a spoken phrase is repeated multiple times*. *After several repetitions*, *the spoken phrase is heard by some listeners as more “song-like” rather than being spoken*. *We want to know if you experience this illusion or not*.

*In this study*, *you will listen to an audio clip of a spoken phrase*. *The phrase will repeat several times*. *At the end of the repetitions*, *please report whether you heard the phrase as being spoken or as being sung*.

After the explanation of the illusion, another screen appeared for 20 seconds informing participants that they would need access to speakers or headphones to listen to the audio file for our study. To encourage participants to attend to the stimulus they were informed that they would need to respond to a question regarding the content of the audio file when the file ended.

When they were ready, participants clicked on a link that opened a new browser tab and automatically played the audio file. The audio file played for 27 seconds, and the tab could not be closed until the allotted time of 30 seconds expired. Participants were then asked several questions about their experience of the Speech-to-Song Illusion: Did you experience the Speech-to-Song Illusion (yes or no)? and What phrase was being repeated in the audio file? The entire survey took less than 5 minutes to complete.

### Results

Of the 199 participants who were recruited and completed the survey, 157 participants responded correctly to the question regarding the content of the audio file, with 115 (73%) of those participants indicating that they experienced the Speech-to-Song Illusion and 42 (27%) indicating that they did not experience the Speech-to-Song Illusion. When considering the musical experience of the participants, 32 indicated that they had no musical experience with 9 (28%) of them reporting that they did not experience the Speech-to-Song Illusion, and 23 (72%) of them reporting that they did experience the Speech-to-Song Illusion. For the 125 participants that reported musical training (with a mean of 12.58 years of musical experience; range from 1 to 65 years of experience), 33 (26%) of them reported that they did not experience the Speech-to-Song Illusion, and 92 (74%) of them reported that they did experience the Speech-to-Song Illusion. The slight difference in the percentage of participants with or with no musical experience who experienced the illusion was not statistically different, as confirmed by a Kolmogorov-Smirnov test (*D* = .5, *p* = 1).

**Fig 2:**
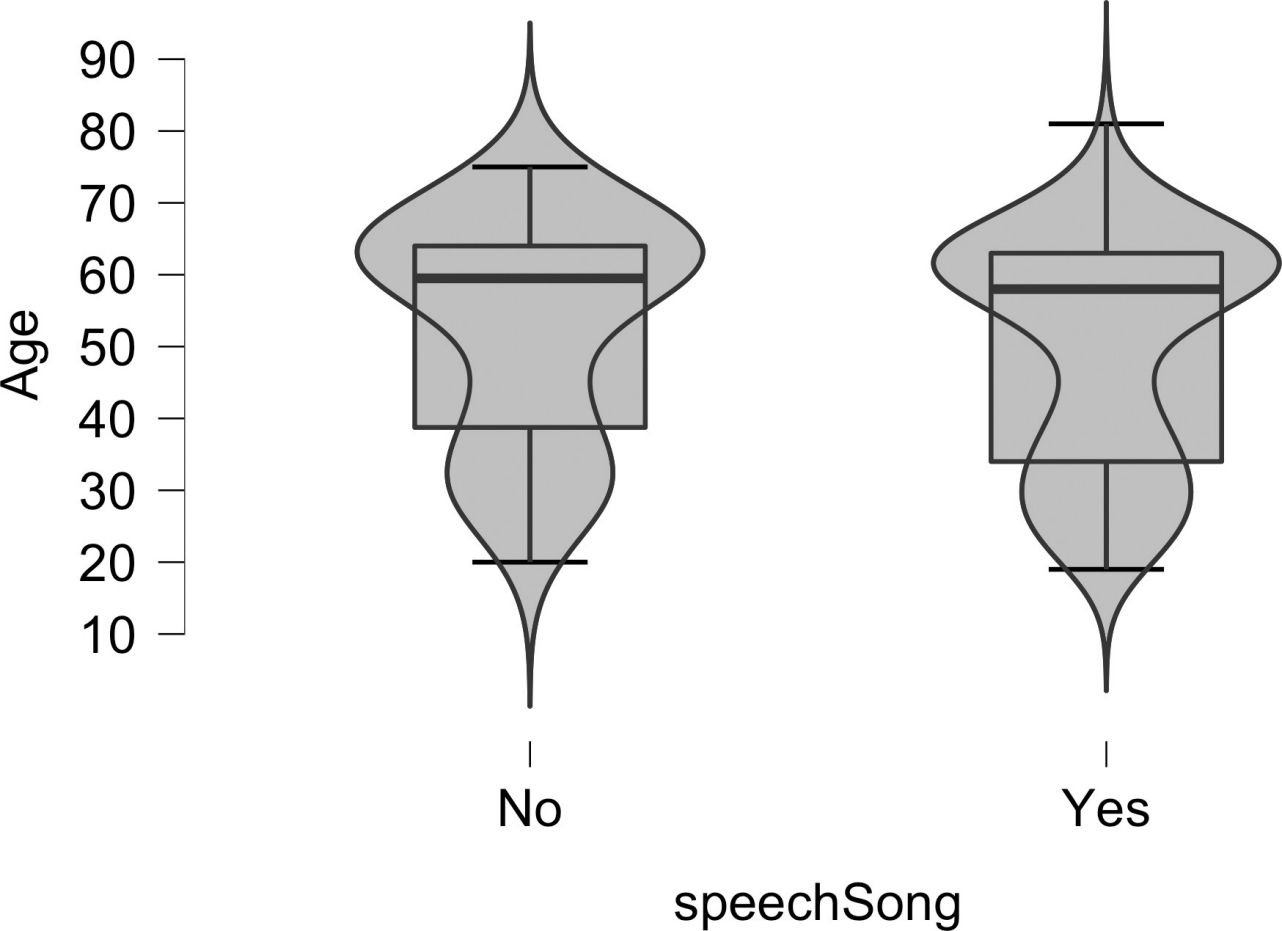
A violin plot with a box plot superimposed of the ages of 157 participants who did not (no) and who did (yes) experience the Speech-to-Song Illusion. The violin plot shows the distribution of ages for each response. The box plot shows the median age (the dark horizontal lines), the 75^th^ to the 25^th^ quartiles (the boxes), and the upper and lower boundaries of 1.5*75^th^ (or 25^th^) quartiles (the whiskers).

Probit regression (using the glm package version 3.4.3 in R Studio Version 1.1.414) was used to determine if the age of participants influenced whether they experienced the Speech-to-Song Illusion (yes or no). Contrary to our predictions, the probit regression analysis was not statistically significant, *probit* (155) = -.01, *p* = .348. In Fig 2 we show violin plots with boxplots superimposed of the ages of participants who did experience the Speech-to-Song Illusion (*n* = 115, *mean* = 50.10 years old; *sd* = 16.13) and who did not experience the Speech-to-Song Illusion (*n* = 42, *mean* = 52.79 years old; *sd* = 15.66).

Given the null result in the probit analysis, we attempted to analyze the data using Bayesian equivalence testing to assess the strength of the evidence for the null hypothesis. Although equivalence testing is common in pharmaceutical research [30], equivalence testing is not as widely used in other areas, such as Psychology [31, 32; see also 8]. For this analysis, we compared the ages (values reported above) of those who experienced the illusion to the ages of those who did not experience the illusion using an Equivalence Bayesian independent samples *t*-test in JASP [33]. The overlapping-hypothesis Bayes factor (BF_∉∈_) was .015 and the nonoverlapping-hypothesis Bayes factor (BF_∈∉_) was 68, which provide strong [34] to very strong [35] evidence that the age of people who experienced the illusion is equivalent to the age of people who did not experience the illusion.

### Discussion

One finding from the present study is that a similar percentage of individuals with musical training and without musical training experienced the speech to song illusion (as confirmed by a K-S test). This is perhaps not surprising given that trained musicians and individuals with no musical training both experience the speech to song illusion [23].

What was surprising in the present study was that age did not influence the experience of the Speech-to-Song Illusion. Although NST accounts for older adults experiencing the TOT state more frequently than younger adults [11], and age effects have been observed in some visual illusions [15, 36] and in the perception of speech [14], we did not observe a difference in the experience of the Speech-to-Song Illusion as a function of age in the present study. Bayesian equivalence testing further indicated that the age of those who experienced the illusion was equivalent to the age of those who did not experience the illusion.

Failing to find a difference in the experience of the Speech-to-Song Illusion as a function of age was not as we predicted. However, the present survey does provide us with a piece of information that had not been reported previously: an estimate of how often the Speech-to-Song Illusion is experienced in a relatively broad and diverse sample of listeners. The result of our survey indicates that 73% of the total 157 participants experienced the illusion. Because this value is not (approaching) 100%, it is unlikely that the value we observed in the present study was influenced by providing participants in the instructions a description of the speech to song illusion (i.e., a conformity bias). In addition, anecdotal reports suggest that visual and auditory stimuli do not evoke illusory percepts in 100% of the population. Finally, it has been reported that 84% of participants rated repetitive words as more song-like in a laboratory-based experiment [7], which is comparable to the value we obtained via an on-line survey. To the best of our knowledge, no other values have been reported regarding the percentage of people one might expect to experience a particular illusion. Despite our age-related prediction not being confirmed in the present survey, obtaining an estimate of how many people in a relatively broad and diverse sample of listeners typically experience the Speech-to-Song Illusion is an important piece of information to have obtained from the present survey.

## Study 2

We considered the possibility that the dichotomous (*i*.*e*., yes or no) response used in Study 1 may have been too coarse-grained a measure for observing any age-related differences that might occur in experiencing the Speech-to-Song Illusion due to the transmission deficit hypothesis affecting the priming of syllable nodes. It is possible that older adults still perceive the Speech-to-Song Illusion (as the results of Study 1 suggest), but perhaps the illusion is weaker in older adults than in younger adults.

To assess the strength of the illusory percept, we did another survey. This time, instead of asking yes/no if the illusion was experienced, we asked participants to rate how song-like the stimulus was on a 1 (sounds like speech) to 5 (sounds like song) scale used in previous studies of the Speech-to-Song Illusion [*e*.*g*., 1, 37]. Given the detrimental influence of the transmission of priming with age (*i*.*e*., the transmission deficit hypothesis) and age effects observed in some visual illusions [15, 36] and in spoken word recognition [14], we predicted that older adults would rate the illusion as less song-like compared to younger adults in the present study.

### Method

#### Participants

We restricted our recruitment on Amazon Mechanical Turk (MTurk) to individuals from the United States of America. A group of 100 participants was initially recruited with no other restrictions. After examining the reported ages of that initial group of participants, we wished to obtain a larger number of older adults, so, we recruited 50 more participants, this time restricted in age to 55 years and older. In total, 150 participants were recruited and completed the survey. Participants were paid $0.25 for their time.

To screen out bots, individuals who did not attend to the task, or individuals that may have experienced age-related hearing problems that adversely affected speech perception, we excluded participants who responded incorrectly to a question regarding the content of the audio file (*i*.*e*., What phrase was being repeated in the audio file?). This left data from 126 participants ranging in age from 19–77 years to be analyzed. The mean age of all of the participants was 46.83 years (*sd* = 16.43).

#### Stimuli

The same stimulus used in Study 1 was used in the present survey.

#### Procedure

The procedure in the present survey was the same as in Study 1, with the exception of asking for a rating on a 5-point scale (1 = sounds like speech, 5 = sounds like song), instead of asking for a yes/no response. The present study was also approved by the institutional review board at the University of Kansas.

### Results

**Fig 3:**
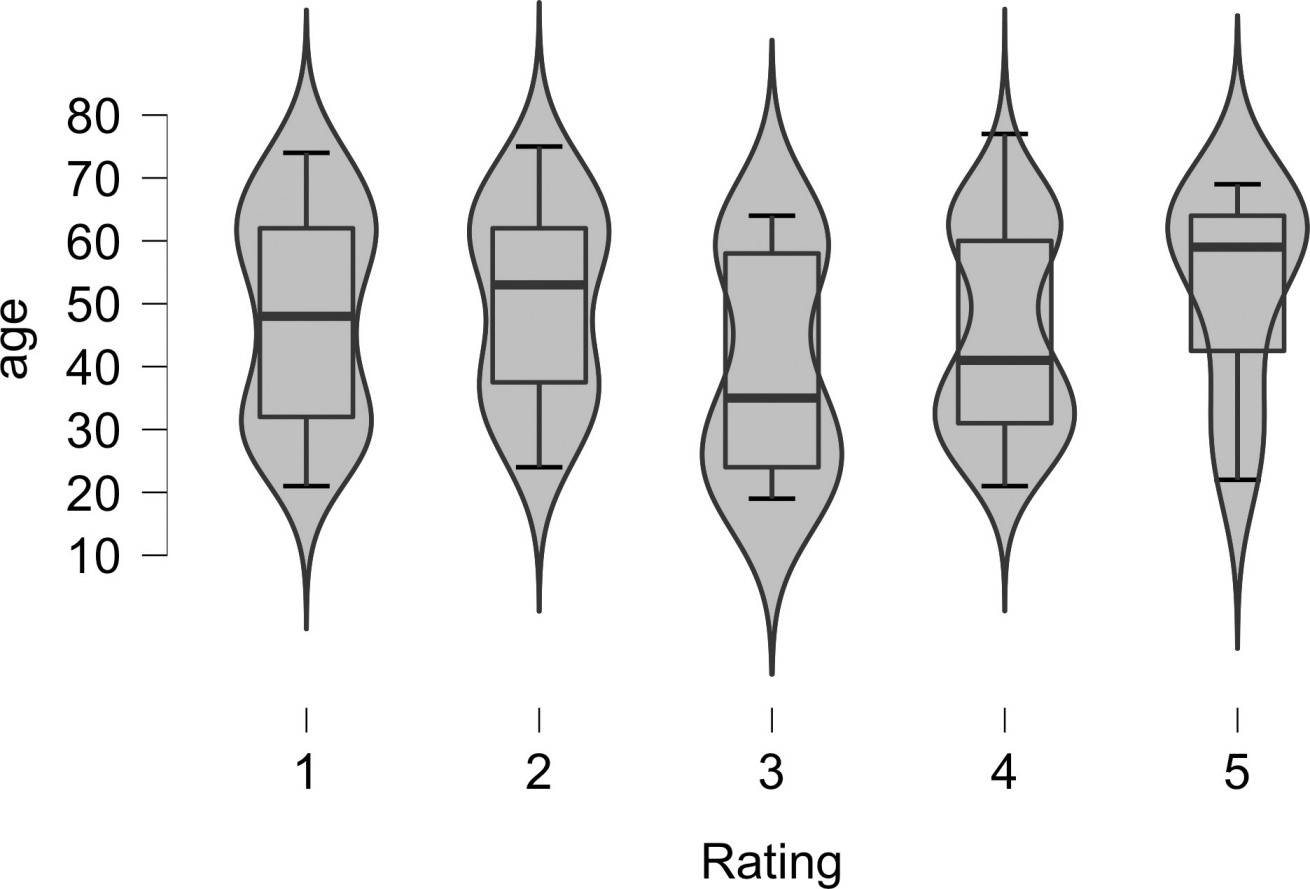
Violin plots with box plots superimposed of the ages for each song rating (1 = sounds like speech, 5 = sounds like song) for 126 participants. The violin plot shows the distribution of ages for each rating. The box plot shows the median age (the dark horizontal lines), the 75^th^ to the 25^th^ quartiles (the boxes), and the upper and lower boundaries of 1.5*75^th^ (or 25^th^) quartiles (the whiskers).

Of the 150 participants who were recruited and completed the survey, 126 participants responded correctly to the question regarding the content of the audio file and were included in the analyses. Given that the rating scale is ordinal, a simple correlation analysis in JASP [33] was used to determine if the age of participants was related to their song-likeness rating on a 5-point scale (1 = sounds like speech, 5 = sounds like song). Contrary to our predictions, the correlation was not statistically significant (Pearson’s *r* = -0.04, *p* = .65). A Bayesian correlation in JASP [33] obtained a Pearson’s *r* = -.04 with a Bayes Factor (BF_10_) of .12, which is considered moderate evidence for H_0_ [38]. In Fig 3 we show violin plots with boxplots superimposed of the ages of participants for each of the song-likeness ratings.

To analyze the data in a different way, we considered the boundary between song and speech placed at the rating of 3 that is shown in Fig 2 (but not discussed anywhere in the text) of [37]. Of the 126 participants in the present study, there were 75 (60%) who reported a 3 or higher on the rating scale and therefore might be considered to have “experienced” the shift from speech to song. The correlation between age and rating in this subset of participants was not statistically significant (Pearson’s *r* = .22, *p* > .05). A Bayesian correlation obtained a Bayes Factor (BF_10_) of .84, which is considered anecdotal evidence for H_0_ [38].

Considering now the possible influence of musical experience, 24 participants reported no musical experience (and were assigned a value of 0 years of experience in this analysis), and 102 participants reported musical experience ranging from 1 to 60 years (*mean* = 10.84 year). A linear regression in JASP using age and years of musical experience to predict the speech to song rating showed that neither age (standardized *b* = -.04, *t* (123) = -0.43, *p* = .66) nor years of musical experience (standardized *b* = -0.001, *t* (123) = -0.015, *p* = .99) predicted the speech to song rating.

### Discussion

Although it seemed reasonable to predict an age-related difference in how often (Study 1) or in the strength with which the Speech-to-Song Illusion (the present study) is experienced—based on the age effects that have been observed in some visual illusions [15, 36] and in speech perception [14], and on the account of TOT states in NST [11]—we did not observe in the present survey a relationship between age and song-likeness ratings. As in Study 1, the result of the present survey was not as we predicted.

## Study 3

To examine possible effects of age on the Speech-to-Song Illusion, we examined in Study 1 the extent to which participants varying in age reported experiencing the illusion. We predicted that more younger participants would report experiencing the illusion than older participants. However, the percentage of participants that reported experiencing the illusion did not differ significantly with age (or musical experience). In Study 2, instead of using a coarse-grained yes/no response as in Study 1, we used a more fine-grained, 5-point rating scale typically used in studies of the Speech-to-Song Illusion to examine if the strength of experiencing the illusion varied with age. We predicted in Study 2 that younger participants would have higher ratings on the song-likeness scale than older participants. However, there again was no significant difference in ratings as a function of age (or musical experience).

In Study 3 we examined “when” listeners varying in age experienced the Speech-to-Song Illusion. In the present task, participants heard the same repeated phrase that was used in Studies 1 and 2, but this time they were asked to click on a button as soon as they experienced the perceptual shift from speech to song, or to click a different button if they did not experience the illusion. Given that the transmission deficit in older adults results in older adults having more difficulty transmitting priming between nodes, leading to impaired activation of certain nodes [13], perhaps more time must pass in order for sufficient amounts of priming to accrue and be transmitted to the syllable nodes—which encode the rhythmic information of language and are thought to lead to the song-like percept in the Speech-to-Song Illusion. In that case, younger listeners may experience the perceptual shift from speech to song sooner than older listeners as measured (in seconds) by the time-to-click.

### Method

#### Participants

To recruit older adults, we restricted our recruitment on Amazon Mechanical Turk (MTurk) to individuals from the United States of America who were 55 years of age or older. Older participants were paid $0.25 for their time. Due to financial constraints, we recruited younger adults who were undergraduate students at the University of Kansas enrolled in Introduction to Psychology to complete the same on-line survey that was presented to the MTurk participants. The participants recruited from the University of Kansas received course credit for their participation in the study.

To screen out bots, individuals who did not attend to the task, or individuals that may have experienced age-related hearing problems that adversely affected speech perception, we excluded participants who responded incorrectly to a question regarding the content of the audio file (*i*.*e*., What phrase was being repeated in the audio file?). This left data from 153 participants recruited via mTurk ranging in age from 56–77 years, and 205 participants recruited via SONA ranging in age from 18–26 years to be analyzed. The mean age of all of the participants was 39.15 years (*sd* = 22.23).

#### Stimuli

The same stimulus used in Studies 1 and 2 was used in the present survey.

#### Procedure

The present study was approved by the institutional review board at the University of Kansas. The procedure in the present survey was the same as in Studies 1 and 2, with the exception of participants receiving the instructions below for the slightly different task:

*Illusions occur when we incorrectly perceive what is in our environment*. *In this study*, *we are interested in an auditory illusion called the Speech-to-Song Illusion*. *In the Speech-to-Song Illusion*, *a spoken phrase is repeated multiple times*. *After several repetitions*, *the spoken phrase is heard by some listeners as more “song-like” rather than being spoken*. *We want to know when you experience this perceptual shift*.

*In this study*, *you will listen to an audio clip of a spoken phrase*. *The phrase will repeat several times*.

*If the phrase shifts from speech to song please click the button labeled "I experienced the illusion" as soon as the shift occurs*.

*If you do not experience this illusion (not all people do) then simply wait until the sound file is done playing and then click the button labeled "I did NOT experience the illusion*.*"*

### Results

Of the 153 participants who were recruited via mTurk, completed the survey, and responded correctly to the question regarding the content of the audio file, 47 reported that they did not experience the illusion (31%), and 106 indicated that they did experience illusion (69%). Of the 205 participants who were recruited via SONA, completed the survey, and responded correctly to the question regarding the content of the audio file, 81 reported that they did not experience the illusion (40%), and 124 indicated that they did experience illusion (60%). The slight difference across the two age groups in the percentage of participants who indicated that they experienced the illusion was not statistically different, as confirmed by a Kolmogorov-Smirnov test (*D* = 1 *p* > .05). Only the time-to-click from participants who indicated that they experienced the illusion were analyzed further.

A traditional and a Bayesian ANOVA in JASP [33] were used to determine if the age of participants and their musical experience influenced time-to-click, which indicated when they experienced the perceptual shift from speech to song. Contrary to our predictions, the main effect of age was not statistically significant in the traditional ANOVA (*F (1*, *226)* = .29, *p* = .59), and obtained a Bayes Factor (BF_10_) of .15, which is considered moderate evidence for H_0_ [38]. The main effect of musical experience was not statistically significant in the traditional ANOVA (*F (1*, *226)* = 1.37, *p* = .24), and obtained a Bayes Factor (BF_10_) of .32, which is considered moderate evidence for H_0_ [38].

The interaction of age and musical experience also was not statistically significant in the traditional ANOVA (*F (1*, *226)* = .41, *p* = .52). The Bayesian ANOVA model with a main effect of age, a main effect of musical experience, and an interaction between age and musical experience obtained a Bayes Factor (BF_10_) of .01, which is considered extreme evidence for H_0_ [38].

The mean time-to-click for the (older) participants recruited via mTurk who reported musical experience was 13.58 seconds (*sd* = 7.25; *n* = 85), and was 15.77 seconds (*sd* = 8.74; *n* = 21) for those who reported no musical experience. The mean time-to-click for the (younger) participants recruited via SONA who reported musical experience was 13.71 seconds (*sd* = 6.85; *n* = 99), and was 14.35 seconds (*sd* = 8.01; *n* = 25) for those who reported no musical experience.

**Fig 4:**
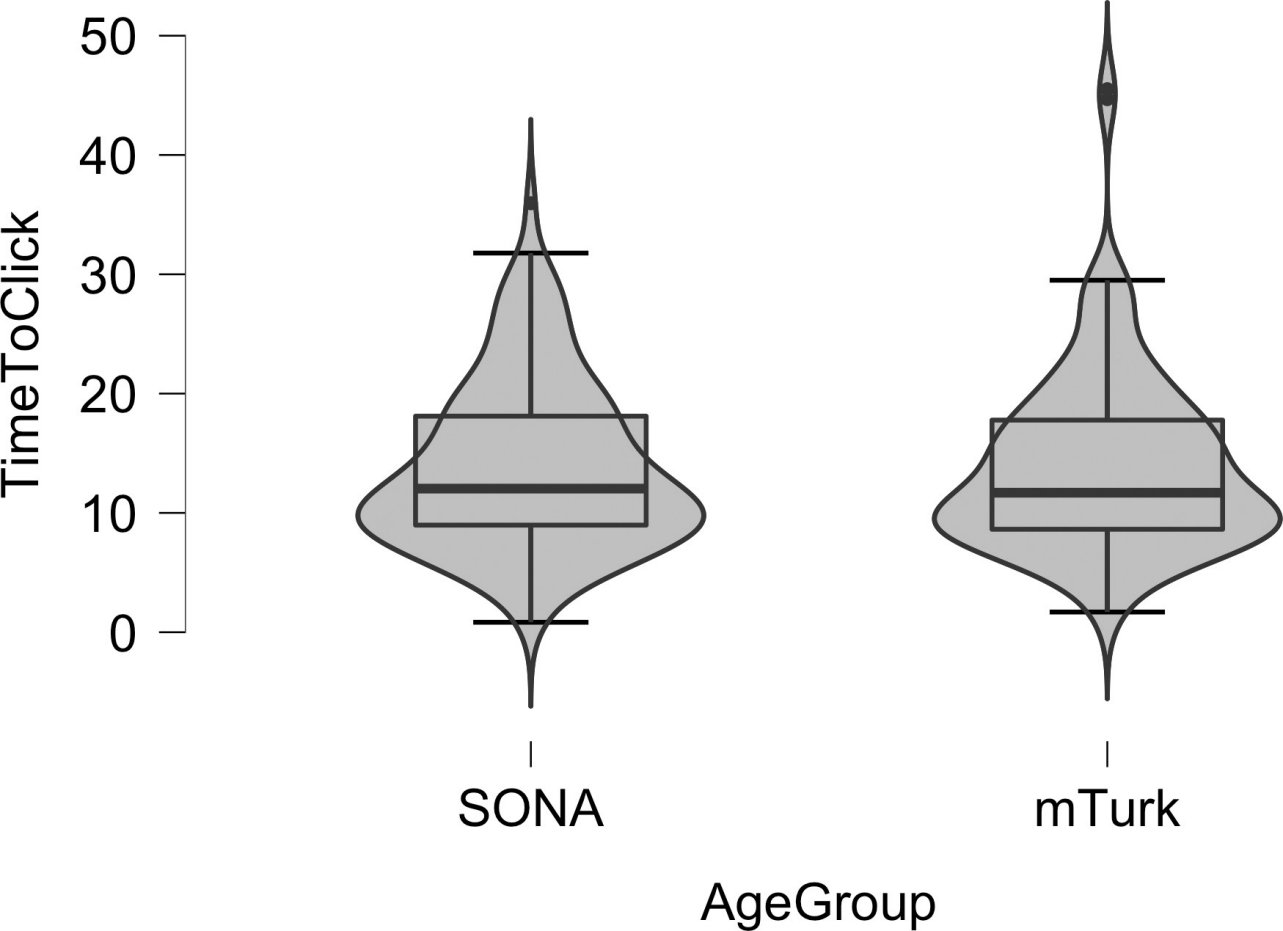
Violin plots with box plots superimposed of the time-to-click for the 124 younger participants (recruited via SONA) and the 106 older participants (recruited via mTurk) who indicated that they experienced the speech to song illusion. The violin plot shows the distribution of ages for time-to-click. The box plot shows the median age (the dark horizontal lines), the 75^th^ to the 25^th^ quartiles (the boxes), and the upper and lower boundaries of 1.5*75^th^ (or 25^th^) quartiles (the whiskers). Individual dots indicate outliers.

In Fig 4 we show violin plots with box plots superimposed of the ages of participants and the time-to-click. As can be seen in the figure and as is common for reaction time distributions, the time-to-click distribution for the younger participants (recruited via SONA) was not normally distributed, as indicated by a significant Shapiro-Wilks test in JASP (*W* = .947, *p* < .001). Similarly, the time-to-click distribution for the older participants (recruited via mTurk) was also not normally distributed, as indicated by a significant Shapiro-Wilks test (*W* = .883, *p* < .001).

### Discussion

In Study 3, we used time-to-click on a button to indicate when listeners varying in age experienced the perceptual shift from speech to song that occurs in the Speech-to-Song Illusion. Given the difficulty in the transmission of priming between nodes that occurs with age (i.e., the transmission deficit hypothesis [13]), we predicted that older adults would need more time than younger adults in order for the perceptual shift to occur, because older adults would need sufficient amounts of priming to accrue and be transmitted to the syllable nodes (which encode the rhythmic information of language and are thought to lead to the song-like percept in the Speech-to-Song Illusion). However, the time-to-click did not differ significantly as a function of age. Both younger and older adults experienced the perceptual shift from speech to song after about 14 seconds of hearing the repeating phrase. That duration corresponds to 5 complete repetitions of the phrase “sometimes behave so strangely” in the 27-second-long sound file used in the three studies reported here. Similar to Studies 1 and 2, musical experience also did not influence when participants experienced the perceptual shift from speech to song that occurs in the Speech-to-Song Illusion.

Although age effects have been observed in some visual illusions [15, 36] and in speech perception [14], the present results suggest that the Speech-to-Song Illusion is experienced similarly across the lifespan. As in Studies 1 and 2, the result of the present survey was not as we predicted.

## General discussion

The results of three surveys failed to support our hypothesis of an age-related difference in experiencing the Speech-to-Song Illusion. Despite the failure to confirm our hypothesis, we did obtain estimates of how many people across a wide range of ages might be expected to experience the Speech-to-Song Illusion that were consistent across the studies despite different measures being used in each survey. The estimates obtained in the three surveys (Study 1 = 73%; Study 2 = 60%; Study 3 = 60–69%) are similar to the value obtained by [7], who reported that 84% of their participants rated repetitive speech as more song-like than non-repetitive speech in a laboratory-based experiment that used more than the 1 stimulus that was used in the present studies. Aside from anecdotal reports, the value reported in [7], and the present study, there are few if any estimates of how often a stimulus might be expected to elicit an illusory percept in the general population.

In addition, the results of Study 3 provide an estimate of when the perceptual shift from speech to song occurs in the speech to song illusion; after about 14 seconds or 5 complete repetitions of the phrase. Note that [16] observed that the speech to song illusion emerged most frequently after 3 repetitions of a sentence in German. They further reported in their Experiment 1 that with alterations to the pitch or rhythmic properties of the stimulus the emergence of the speech to song illusion occurred after the 4^th^ or 5^th^ repetition. Thus, the observed value of 5 repetitions for the speech to song illusion to emerge in the present study is comparable to previously observed values regarding the number of repetitions required to shift the percept from speech to song.

Future studies will need to determine if it is the amount of time exposed to the stimulus or the number of repetitions of the stimulus phrase that is the more crucial contributor to the phenomenological experience of the speech to song illusion. The present data are not able to distinguish between the two possibilities. Examining the role of the amount of time exposed to the stimulus and the number of repetitions of the stimulus phrase in experiencing the speech to song illusion may also help determine if a similar illusion—the sound to song illusion [39]—is governed by the same mechanisms that lead to the speech to song illusion, or if different cognitive mechanisms contribute to these auditory illusions that appear superficially similar.

We acknowledge the limitations that the “one-shot” method employed in the present surveys may impose on investigating an age-related influence on the perception of the Speech-to-Song Illusion. Our desire to obtain a large sample with a wide range of ages led us to recruit participants through MTurk, prompting us to use a very simple, streamlined presentation of one stimulus/one trial in order to minimize the time demands on participants. Despite reasonably large sample sizes in the studies, it is possible that the one-shot method (using a phrase that is likely to evoke the illusion) did not provide enough data to assess an age-related difference in perceiving the Speech-to-Song Illusion. Perhaps presenting more trials in a traditional laboratory-based setting (as in [7]) might increase our sensitivity to detect an age-related difference, if it exists. For example, if an age-related difference exists, one might expect that younger adults might experience the illusion on 10 out of 10 trials, whereas older adults might experience the illusion on 7 out of 10 trials.

Although there are many studies that demonstrate age-related declines in various aspects of cognitive performance, there are a number of studies that indicate that age-related declines do not affect all aspects of cognitive performance. A large body of work was reviewed by [40] and suggests that certain aspects of speech production tend to be affected by age, but many aspects of speech comprehension remain relatively stable and unaffected by age. Perhaps the Speech-to-Song Illusion is one of those cognitive phenomena that remains relatively stable and unaffected by increased age.

Alternatively, instead of looking for an age-related difference in perceiving the Speech-to-Song Illusion among older adults, perhaps we may need to consider the other end of the developmental spectrum and look at much younger individuals. Consider the development of the visual perception and locomotion systems in infants that affect performance in the visual cliff (e.g., [35]). Also consider the protracted development of spatial integration skills that influence whether various visual illusions are perceived by four-year-olds, seven-year-olds, and adults [15]. If NST, a model of language processing, does provide an adequate account of the Speech-to-Song Illusion, and there is an age-related difference in the perception of this auditory illusion, perhaps we need to look for that difference in infants before the language system has fully developed using the head-turn preference procedure commonly used to study language development in infants [41]. Recent research shows that some zoo animals experience certain perceptual illusions [2], suggesting that the mechanisms responsible for certain perceptual illusions may have evolutionarily old origins. Given the later evolutionary emergence of language it might be useful to test whether other animals that do not use language also experience the Speech-to-Song Illusion.

Finally, we recognize that NST is a verbal model of the recognition and production of spoken language, not a computer model capable of generating precise predictions via computer simulations [42]. Given the complexity of this verbal model, it is possible that we may have over-extended the influence that the transmission deficit hypothesis might play in the Speech-to-Song Illusion. We note that the transmission deficit hypothesis has been studied extensively in speech production (e.g., [43, 44]), but to our knowledge, it has been studied much less in the context of speech perception [13], making it unclear the extent to which the transmission deficit hypothesis might affect speech perception or perceptual illusions, like the Speech-to-Song Illusion. Therefore, the present studies—while clearly inspired by the cognitive mechanisms in NST—should be viewed as being somewhat exploratory in nature rather than being a challenge to the mechanisms found in NST, which have been used to account for a wide range of cognitive phenomena (e.g., [10, 11, 13, 45, 46]), including another auditory illusion known as the verbal transformation effect [47, 48].

Although our hypothesis about age affecting the Speech-to-Song Illusion was not supported by the results from the three studies reported here, it is important to continue to investigate perceptual illusions. Perceptual illusions provide psychologists with other ways to examine the limits of perceptual and cognitive systems, potentially increasing our fundamental understanding of these systems [3, 4, 49, 50]. Recent work has demonstrated that song and infant-directed speech facilitates the process of word learning in adults [51]. Because both (a register of) speech and song influence the language-related process of word-learning, further examination of auditory illusions like the Speech-to-Song Illusion might provide insight in to a wide range of other language-related processes.

## Supporting information

S1 Data (ZIP)
